# Quantification of Mesenchymal Stem Cell (MSC) Delivery to a Target Site Using In Vivo Confocal Microscopy

**DOI:** 10.1371/journal.pone.0078145

**Published:** 2013-10-29

**Authors:** Luke J. Mortensen, Oren Levy, Joseph P. Phillips, Tara Stratton, Brian Triana, Juan P. Ruiz, Fangqi Gu, Jeffrey M. Karp, Charles P. Lin

**Affiliations:** 1 Wellman Center for Photomedicine and Center for Systems Biology, Massachusetts General Hospital, Harvard Medical School, Boston, Massachusetts, United States of America; 2 Center For Regenerative Therapeutics & Department of Medicine Brigham and Women’s Hospital, Harvard Medical School, Harvard Stem Cell Institute, Harvard-MIT Division of Health Sciences and Technology, Cambridge, Massachusetts, United States of America; Tufts University, United States of America

## Abstract

The ability to deliver cells to appropriate target tissues is a prerequisite for successful cell-based therapy. To optimize cell therapy it is therefore necessary to develop a robust method of in vivo cell delivery quantification. Here we examine Mesenchymal Stem Cells (MSCs) labeled with a series of 4 membrane dyes from which we select the optimal dye combination for pair-wise comparisons of delivery to inflamed tissue in the mouse ear using confocal fluorescence imaging. The use of an optimized dye pair for simultaneous tracking of two cell populations in the same animal enables quantification of a test population that is referenced to an internal control population, thereby eliminating intra-subject variations and variations in injected cell numbers. Consistent results were obtained even when the administered cell number varied by more than an order of magnitude, demonstrating an ability to neutralize one of the largest sources of in vivo experimental error and to greatly reduce the number of cells required to evaluate cell delivery. With this method, we are able to show a small but significant increase in the delivery of cytokine pre-treated MSCs (TNF-α & IFN-γ) compared to control MSCs. Our results suggest future directions for screening cell strategies using our in vivo cell delivery assay, which may be useful to develop methods to maximize cell therapeutic potential.

## Introduction

Cell-based therapeutics offer the potential to address unmet clinical needs in which traditional health care has faltered. Cellular therapies have been explored in pre-clinical and clinical models, and demonstrated promise in diseases such as lung injury [1], myocardial infarction [2], [3], graft versus host disease [4], [5], and sepsis [6]. However, very few clinical applications have been approved so far, which suggests that treatment efficacy could be improved. One of the primary strategies to improve therapeutic outcome is by increasing delivery of cells to their target tissue. To do so, methods such as alternative culture [7], [8], pretreatment with cytokines [9], [10], [11], transfection [12], [13], [14], or cell engineering [15], [16], [17], [18] have been used. Our lab has primarily focused on cell surface engineering of therapeutic mesenchymal stem cells (MSCs), and has found that functionalization of the MSC surface can enhance their delivery to an inflamed site in vivo [18].

To evaluate the delivery of potential cell therapeutics in vivo, the most common techniques are radiolabeling [19], [20], bioluminescence [21], [22], [23], [24], fluorescent protein expression [25], [26], [27], [28], [29], and exogenous fluorescence labels [17], [18], [30], [31]. Of these, only fluorescent protein expression and exogenous fluorescence labeling have been demonstrated to have adequate sensitivity for single cell detection in vivo. Fluorescent protein expression is a powerful technique when purification of cells from transgenic mice or transfection using lentivirus is possible. However, transfection can yield variable fluorescent protein expression [32], [33] and impact cell function [34], and as such is not optimal for all applications. Therefore, to track cell delivery to inflamed tissues, we stain the cell membrane with lipophilic membrane dyes and image the cells in vivo using confocal microscopy. Single cell detection using confocal microscopy allows dynamic and quantitative tracking of cells in vivo, an important capability in the evaluation of cell modification strategies and elucidation of biological mechanisms. Previously published research by our group and others has demonstrated the usefulness of this strategy to evaluate the impact of cell surface engineering in vivo using MSCs. In particular, studies by Sackstein et al. and Sarkar et al. found that surface engineering of MSCs stained with lipophilic membrane dyes enhanced delivery to the bone marrow via enzymatic modification and to the inflamed ear via Sialyl Lewis^x^ chemical modification, respectively [18], [30].

One significant advantage of fluorescent cell labels is the ability to detect multiple colors at once, a strategy leveraged by Sarkar et al. When combined in an optimized dye pair, simultaneously administered modified and control cells can be quantified, which allows each animal to serve as its own control and limits animal-to-animal variability. The aim of this study is to select the optimal dye pair combination from a series of 4 membrane stains for quantifying cell delivery to inflamed tissue using MSCs by elucidating the practical optical characteristics of each cell tracking dye from visible to near-IR emission. Our results will improve the ability of researchers to quantify and optimize in vivo cell homing behavior.

## Results and Discussion

### In Vitro MSC Staining and Viability

**Figure 1:**
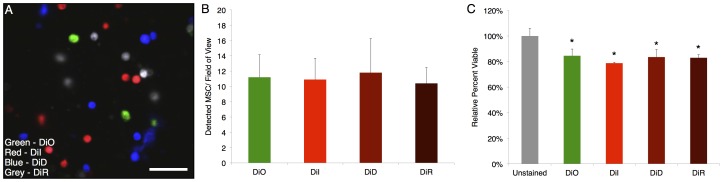
MSC staining in vitro. In vitro imaging characteristics of MSCs stained with lipophilic membrane dyes (a). Mixing equal quantities of the cells yielded similar levels of detected cells (b). Despite a small decrease in viability from the unstained control (p<0.05, Tukey’s HSD), there was minimal difference between viability of cells after staining as measured by MTS (c). Scale bar = 100 µm.

To determine the relative staining efficiency in vitro, stained MSCs were mixed in equal quantities at 10^6^ cells/mL for each color, imaged on a glass slide, and displayed simultaneously (Fig. 1a). In each frame, all MSCs were stained as determined by comparison with the reflectance channel. Quantification of cell numbers (n≈100 for each color, from a total of ∼20 fields of view) shows that about equal number of cells are detected in each color (Fig. 1b). Direct insertion of the dyes into the cell membrane had a limited impact on viability versus the unstained control (p<0.05, Tukey’s HSD) as measured by metabolic activity using MTS with minimal difference between stains (∼80% of unstained for all, Fig. 1c).

### MSC Trafficking to Inflamed Ears

**Figure 2:**
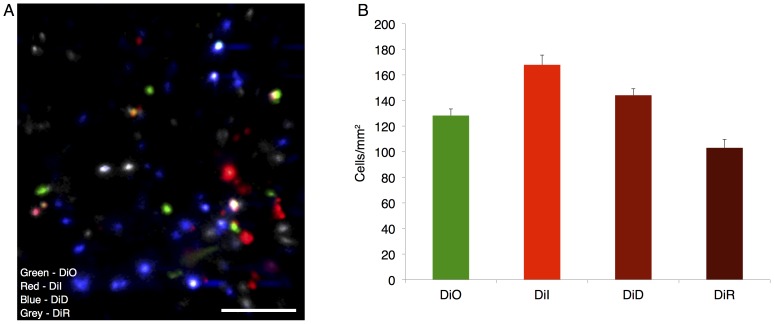
MSC delivery to an inflamed site in vivo. Intravenously infused MSCs stained with a range of lipophilic membrane dyes appear in the inflamed ear 24(a). A stain-dependent difference in detected cell numbers was found (p<0.05, 1-way ANOVA), where DiI and DiD yielded the highest counts in the ear with DiO and DiR having relatively fewer cells detected (b). Scale bar = 100 µm.

To evaluate the performance of lipophilic dyes for use in quantifying cell delivery to an inflamed site, 7×10^4^ MSCs stained with each of the range of dyes were systemically infused. By imaging in the inflamed ear, cells labeled with all four stains can be visualized, as displayed using a maximum intensity projection of the 3D stacks (Fig. 2a). Cell counting was performed on individual channels in the 3D stack with contrast enhanced to enable detection of all events, after first excluding autofluorescent cells which typically showed up as low-intensity objects distributed across than one channels. Upon quantification (Fig. 2b), a difference between the detected number of cells for the stains was observed (p<0.05, 1-way ANOVA). The largest number of MSCs were detected with DiI staining (168±16 cells/mm^2^), followed by DiD (144±11 cells/mm^2^), DiO (128±11 cells/mm^2^) and DiR (103±14 cells/mm^2^). Higher tissue autofluorescence background at shorter wavelengths likely contributed to the lower sensitivity for DiO. Interestingly, the advantages of diminished autofluorescence and better tissue penetration with increasing wavelength did not lead to higher cell counts for DiR, most likely due to the reduced quantum efficiency of our PMTs at wavelengths >700 nm. These results suggest that in terms of detected counts in the inflamed ear, the optimal dye pair for future use is DiI and DiD.

### Depth Dependence of Detection

**Figure 3:**
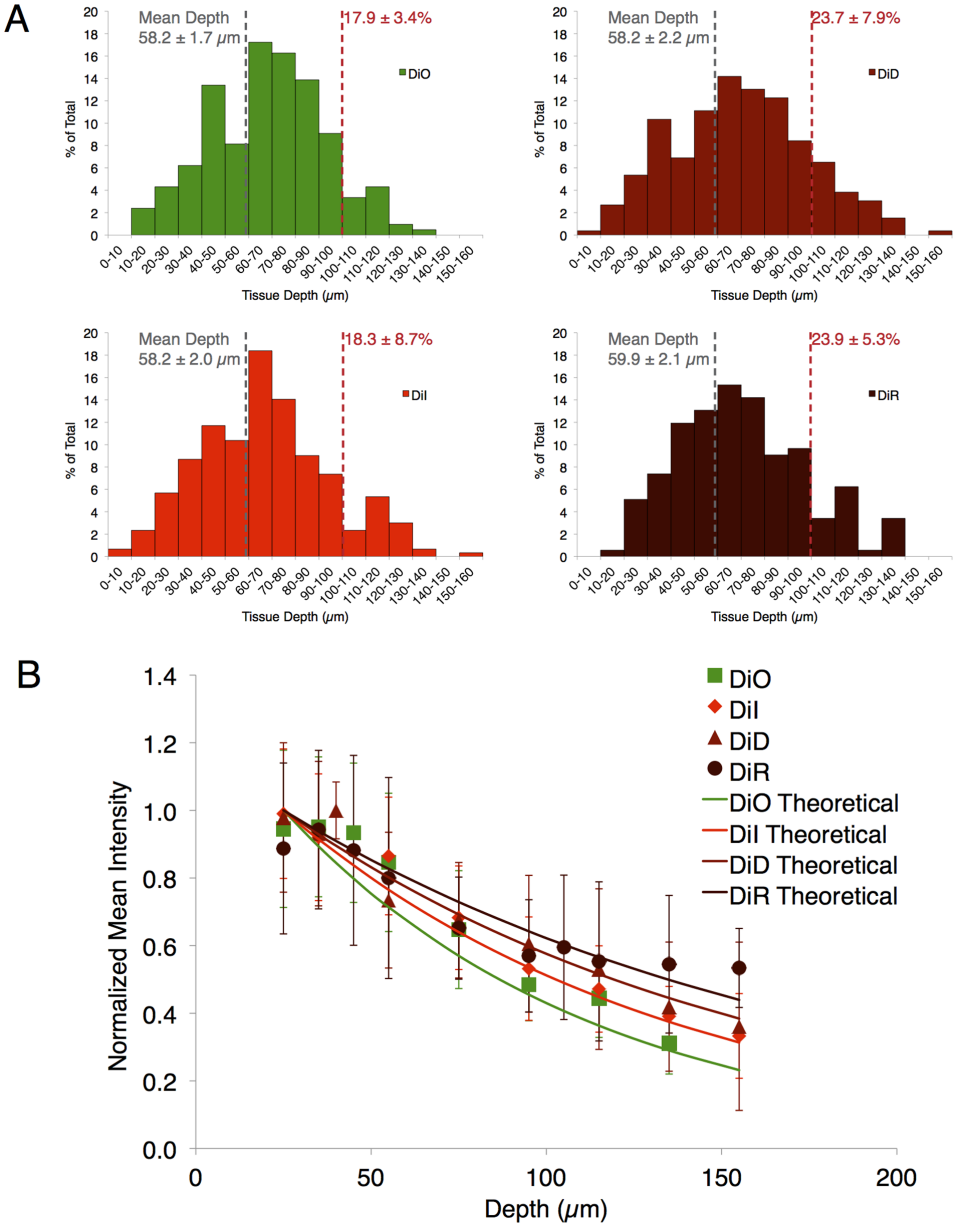
Stained MSC depth detection dependence. When the depth dependence of detection for MSCs dyed with each stain is analyzed, no discernable difference in the depth histogram or in the average MSC depth is observable (a). However, a modest increase in the number of MSCs detectable below the depth of 100 µm appears with longer wavelength dyes. Normalized intensity of cells analyzed over depth indicates a close match between experimental results and theoretical predictions, with an improvement in the intensity retention of the near-IR emitting DiR labeled cells (b). Average signal intensity revealed a lower intensity for DiO- likely due to high background near the skin surface and signal degradation at greater depth- and for DiR. However, DiR intensity was mostly recovered when corrected for detector quantum efficiency, suggesting the importance of detector characteristics in cell detection.

To better characterize how the detection sensitivity varies with wavelength, we next evaluated the depth dependence of detection for the range of MSC stains. This depth distribution of detected cells is a function of the staining intensity, the detection sensitivity of the system at the wavelength of interest, and the scattering properties of the tissue at the excitation/emission wavelengths. Interestingly, little difference can be observed between the cell colors in the depth histogram shape and mean depth (Fig. **3**a). Since our LPS injection is in the subcutaneous space on one side of the ear (mouse ears are ∼200 µm in thickness with collagen support in the center), it is likely that the average depth of detected MSCs of 58 µm is the actual location of the cells. This suggests that the majority of the cells are located at depths sufficiently shallow to be detected regardless of the stain. However, if only the deepest cells are investigated (>100 µm deep), an increase in the proportion of detected cells is observable with increasing dye emission wavelength (Fig. **3**a), which is expected due to the decreased scattering and absorption at longer wavelengths. Additionally, if MSC intensity is examined as a function of depth and normalized to the brightest cells, an increase in intensity for the longest wavelength DiR stained cells is found at the deepest locations (Fig. **3**b). This result is supported by a simple model prediction based on scattering and absorption coefficients reported in the literature for mouse ears with our excitation and peak emission wavelengths [35]. To do so, we used the formula.
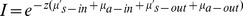
where the relative signal intensity (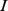) is a function of depth (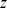, mm), reduced scattering coefficient for excitation (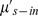, mm^−1^) and emission (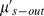, mm^−1^), and absorption coefficient for excitation (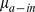, mm^−1^) and emission (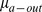, mm^−1^). At a depth of 135 µm, this approximation would suggest a remaining intensity of 29% for DiO, 31% for DiI, 38% for DiD, and 44% for DiR. Our experimental results at 135 µm align well with predicted values (31±9% for DiO, 39±9% for DiI, 42±20% for DiD, and 54±20% for DiR), but with increased error due to the small number of cells that are found at 135 µm depth. These results suggest future work to more carefully investigate contributing factors to accurately model our imaging scheme and determine relative contributions of all optical and electronic parameters [36]. Since DiI and DiD exhibited the highest intensity and counts, this dye pair was selected for subsequent experimentation.

### Detection of Increased MSC Homing with Cytokine Pre-treatment

**Figure 4:**
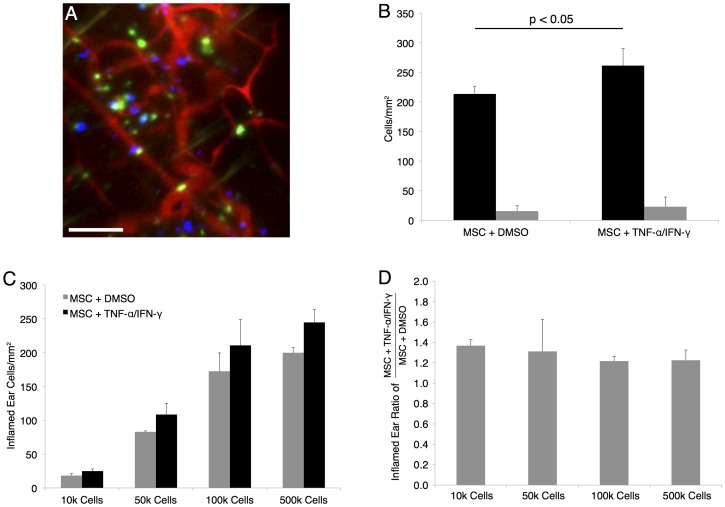
An optimized dye pair allows quantification of MSC cytokine pre-treatment impact on delivery to an inflamed site. Systemic infusion of MSCs pretreated with TNF-α/IFN-γ (blue, stained with Vybrant DiD) increases cell trafficking to the inflamed ear vs. control MSCs (green, stained with Vybrant DiI) (a). When quantified, this yields an average increase in cells/mm^2^ of ear surface area of 22±2% (p<0.05, Students unpaired t-test) (b). The increase in trafficking is of a consistent magnitude across a range of injected cell numbers with a commensurate range of detected counts (p<0.05, 2-way ANOVA) (c). If the increase in homing for each cell dose is plotted, consistent evaluation of the homing increase is observed, even with a wide range of injected cell numbers (d). Scale bar = 100 µm.

One strategy to increase the delivery of MSCs to diseased sites is to pre-treat the cells with a small molecule or cytokine that will up-regulate or activate homing mechanisms of the MSC. As a proof of concept to demonstrate the usefulness of our optimized DiD/DiI dye pair in evaluating MSC homing to the inflamed ear, a cytokine cocktail of TNF-α & IFN-γ was used. Both molecules are well accepted to activate MSC function and homing [37], [38], [39], [40], and were introduced to the cell media in vitro 24 h before infusion (10 ng/mL each). Use of a dye switch with correction for the dye efficiency as described in the materials and methods section allowed direct comparison between the numbers of MSCs detected in the inflamed ear. As shown in Fig. 4a,b, a statistically significant increase of 22±2% was found in the delivery of TNF-α/IFN-γ -treated MSCs to the inflamed site as compared to the control MSCs (p<0.05, unpaired Students t-test). The effect remained with a commensurate reduction in detected cell counts (p<0.05, 2- way ANOVA) when progressively fewer cells were systemically infused from 5×10^5^ cells down to 1×10^4^ cells, which is highlighted by plotting the average ratio of pre-treated MSCs to control MSCs in the inflamed ear at each dose (Fig. 4c,d). An increase in cell delivery this small would likely remain undetected if only a single cell population was analyzed per mouse (i.e. without the co-infusion of treated and control cells labeled with the DiI/DiD dye pair) due to variability among individual animals that could result from parameters such as cell infusion number and the severity of inflammation. These results indicate the power of our technique to neutralize one of the largest sources of in vivo experimentation variability, and build on a previous report demonstrating a larger enhancement of MSC trafficking to inflamed sites [18]. Additionally, validating the injection of significantly fewer cells than the 1×10^6^ per mouse or more commonly used to analyze homing behavior, substantially reduces experimentation burden allows more efficient testing of cell delivery strategies, and more closely mimics cell loading used in human clinical trials [10], [17], [18], [19], [21], [28], [41]. To further increase the throughput potential of our validated inflamed ear quantification technique, future work will pursue automation of cell counting by tailoring techniques described in published cell counting studies [42], [43], [44] to the high background imaging environment of the skin.

## Conclusions

This work demonstrates an optimized corrected dye pair to generate robust quantification of cell delivery to target the inflamed mouse ear using primary human MSCs. We have shown that a range of lipophilic membrane dyes yield consistent staining in vitro and in vivo, and have minimal impact on viability in vitro. We have determined that the bulk of MSCs detected in the dermal layers of the skin appear to be within 55–60 µm of depth, that longer wavelength dyes allow detection of higher numbers of cells deep in the tissue, and the optimal nature of the DiI/DiD dye pair in our system. To apply our findings, we demonstrated increased homing that was consistent across cell injection number for MSCs pretreated with TNF-α/IFN-γ, with use of a dye ratio to correct for injection number and provide a robust quantification of homing improvement. Our optimized assay was able to detect a small increase in cell delivery, and could improve experimental throughput in the future by substantially reducing the number of cells needed for investigation of cell engineering strategies. This knowledge can be used to determine the efficacy of further cell engineering approaches to improve cell trafficking to target tissue, and may help yield novel therapeutics and cell delivery strategies in the future.

## Materials and Methods

### Cell Staining and Viability

To allow tracking of MSCs, cells were stained with a range of lipophilic membrane dyes with emission wavelengths in the green (DiO, extinction coefficient = 154,000 cm^−1^M^−1^ in MeOH), red (DiI, extinction coefficient = 148,000 cm^−1^M^−1^ in MeOH), far red (DiD, extinction coefficient = 193,000 cm^−1^M^−1^ in MeOH), and near-IR (DiR, extinction coefficient = 270,000 cm^−1^M^−1^ in MeOH) (Invitrogen, Carlsbad, CA). Primary human MSCs were suspended at a concentration of 10^6^ cells/mL and incubated with 10 µM Vybrant DiO, 10 µM Vybrant DiI, 10 µM Vybrant DiD, or 15 µM Vybrant DiR in 1×PBS+0.1% BSA for 20 minutes at 37°C. The MSCs were then washed twice in 1×PBS and mixed in equal numbers for imaging in vitro or in vivo at a concentration of 1×10^7^ cells/mL To investigate the impact of lipophilic membrane stains on MSC health, cell viability was tested using the metabolic activity stain MTS according to standard protocols. Equal numbers of the stained MSCs were placed in individual wells in triplicate and incubated 24 h in media at 37°C. The formazan-based reagent was added for 4 h and viability was assessed by absorbance at 590 nm.

### In Vivo MSC Homing

C57BL/6 mice (Charles River Laboratories, Wilmington, MA) were anesthetized with ketamine/xylazine and their ears shaved 24 h prior to cell infusion. To induce an inflammatory response, 30 µg of E. coli lipopolysaccharide (LPS, Sigma, St. Louis, MO) in 50 µL saline was injected into the pinna of the left ear, with 50 µL 0.9% saline injected into the right ear as a control. For in vivo dye sensitivity validation, 70,000 cells of each stain were suspended in 150 µL PBS (pH 7.4) and injected by retro-orbital vein infusion. Studies were in accordance with US National Institutes of Health guidelines for care and use of animals under approval of the Institutional Animal Care and Use Committees of Massachusetts General Hospital and Harvard Medical School.

To evaluate the impact of cytokine pre-treatment of MSC homing to the inflamed ear, MSCs were incubated with 10 ng/mL each of TNF-α and IFN-Υ for 24 h before staining and in vivo administration. Each mouse (n = 4) received a range of cell doses (1×10^4^, 5×10^4^, 1×10^5^, or 5×10^5^) each of cytokine pre-treated cells and control cells stained with DiI or DiD with a dye switch. To highlight the vasculature, 50 µL of 10 mg/mL FITC-dextran (2×10^6^ kDa; Sigma, St. Louis, MO) was injected retro-orbitally prior to imaging.

### Confocal Fluorescence Microscopy

In vitro staining and in vivo homing of stained MSCs to the skin was imaged noninvasively in real time using a custom-built video-rate laser-scanning confocal microscope [45]. For this work, the microscope assembly was expanded to allow the acquisition of 4 fluorescence confocal channels simultaneously. To image cell staining efficiency in vitro, equal numbers of MSCs stained with each of the dyes were mixed and placed on a microscope slide with a coverslip at a concentration of 10^6^ cells/mL. For in vivo imaging, the mouse ear was positioned under a coverslip with methylcellulose gel and images acquired at 30 frames per second at a depth up to 200 µm using a 60X 1.0NA water immersion objective lens (Olympus, Center Valley, PA). DiO labeled MSCs were excited with a 491 nm continuous wave (CW) laser (Cobalt, Stockholm, Sweden), and detected through a 520±20 nm bandpass filter (Semrock, Inc., Rochester, NY). DiI labeled MSCs were excited with a 561 nm CW laser (Coherent, Inc., Santa Clara, CA) and detected through a 593 nm ±40 nm filter (Omega Optical, Brattleboro, VT). DiD labeled MSCs were excited with a 638 nm CW laser (Coherent, Inc., Santa Clara, CA) and detected through a 695 nm ±27.5 nm band pass filter (Omega Optical, Brattleboro, VT). DiR labeled MSCs were excited using a femtosecond Ti:Sapphire Maitai source for single photon excitation at 750 nm (Spectra Physics, Santa Clara, CA) and collected through a 785 nm ±31 nm band pass filter (Omega Optical, Brattleboro, VT).

### Quantification with Dye Switch

For quantification, the average number of cells in 20 representative imaging locations across the inflamed region were counted in each mouse. Cells were defined as having a diameter from 10–30 µm to eliminate debris and clumps from analysis, and a primary channel intensity greater than 2 to eliminate autofluorescent events. To ensure accurate quantification of MSC homing to the inflamed ear, the optimal dye pair was selected as described above and used with a dye switch. In this, half of the test animals received a solution containing DiI control cells and DiD TNF-α/IFN-γ treated cells. The other half of the test animals receive a solution containing DiD control cells and DiI TNF-α/IFN-γ cells. The counts for DiD cells were adjusted by the relative detection sensitivity (ratio of DiI/DiD counts) determined in the overall dye efficiency experiments (Fig. 2b). This dye switch allowed direct comparison of cell homing numbers and ensured that equalization did not introduce bias into our measurements.

### Statistical Analysis

For individual pairwise comparisons, an unpaired two-tailed Student’s t-test was used. For group analyses, 1-way or 2-way ANOVA was used, with Tukey’s HSD to evaluate multiple pairwise comparisons. Error bars in all graphs represent standard deviation, and statistical significance is denoted by *p<0.05.
